# Effect of sera from elite athletes on cytokine secretion and insulin signaling in preadipocytes and skeletal muscle cells

**DOI:** 10.3389/fmolb.2022.943034

**Published:** 2022-11-24

**Authors:** Sara Alheidous, Shamma Al-Muraikhy, Nasser Rizk, Maha Sellami, Francesco Donati, Francesco Botre, Layla Al-Mansoori, Mohamed A. Elrayess

**Affiliations:** ^1^ Biomedical Research Center, Qatar University, Doha, Qatar; ^2^ Biomedical Sciences Department, College of Health Sciences, QU Health, Qatar University, Doha, Qatar; ^3^ Physical Education Department (PE), College of Education, Qatar University, Doha, Qatar; ^4^ Federazione Medico Sportiva Italiana (FMSI), Rome, Italy; ^5^ REDs-Research and Expertise in AntiDoping Sciences, University of Lausanne, Lausanne, Switzerland; ^6^ College of Pharmacy, QU Health, Qatar University, Doha, Qatar

**Keywords:** elite athletes, insulin signaling, inflammation, preadipocytes, skeletal muscle cells, high-endurance sports, high-power sports

## Abstract

**Introduction:** The immunomodulatory effect of physical activity can impact insulin signaling differentially in adipose tissues and skeletal muscle cells, depending on sport intensity. In this study, the effect of serum from elite athletes with varying endurance levels and playing different power sports on cytokine secretion and insulin signaling in preadipocyte and skeletal muscle cell lines was investigated.

**Methods:** Preadipocytes (3T3-L1) and skeletal muscle cells (C2C12) were cultured in media containing pooled sera from elite athletes who play high-endurance (HE), high-power (HP), or low-endurance/low-power (LE/LP) sports for 72 h. Secreted cytokines (IL-6 and TNF-alpha) were assessed in the supernatant, and insulin signaling phosphoproteins levels were measured in lysates following treatment using cells multiplex immunoassays.

**Results:** Sera from LE/LP and HP induced TNF-α secretion in C2C12, while serum from HE reduced IL-6 secretion compared to non-athlete serum control. All elite athlete sera groups caused decreased insulin sensitivity in 3T3-L1 cells, whereas in C2C12 cells, only HE athlete serum reduced insulin signaling, while LE/LP and HP caused increased insulin sensitivity.

**Conclusion:** Sera from elite athletes of different sport disciplines can affect the inflammatory status and insulin signaling of preadipocytes and myoblasts differently, with risk of developing insulin resistance. Furthermore, investigation of the functional relevance of these effects on exercise physiology and pathophysiology is warranted.

## Introduction

Disturbance of insulin signaling leads to insulin resistance (IR), a condition where cells, primarily within the insulin-sensitive tissues, lose their functional response to insulin. This can be caused by different mechanisms such as inflammation and oxidative stress (Boucher et al., 2014). The metabolic consequences of IR, including hyperglycemia, inflammatory marker elevation, and visceral adiposity, might develop into type-2 diabetes mellitus (DM-2) and other chronic inflammatory diseases (Freeman and Pennings, 2022). Inflammation is one of the mechanisms that induce IR through the induction of several pro-inflammatory mediators such as tumor necrosis factor (TNF)-α and interleukin (IL)-1 beta (IL-1β) (Ozes et al., 2001). IL-6 exhibits a dual inflammatory effect, mainly anti-inflammatory in the context of acute-phase myokines (such as in acute muscular contraction) but pro-inflammatory in case of chronic inflammation/contraction (Nara and Watanabe, 2021).

The beneficial effect of exercise on glucose homeostasis is well established in which exercise increases cellular glucose uptake, attributed mainly to enhanced insulin signaling. The exercise can improve insulin signaling directly by enhancing the post-receptor signaling molecules, such as insulin receptor substrate (IRS)-1, IRS-2, and PI3K, or indirectly through modulation of the IR pathophysiological process mainly by exhibiting an anti-inflammatory effect (Yaribeygi et al., 2019). Studies have shown that several hours of acute exercise can trigger a prolonged rise in insulin signaling lasting for up to 72 h post-exercise (DiMenna and Arad, 2021).

While physical inactivity promotes adipose tissue to secrete pro-inflammatory cytokines known as adipokines, exercise induces several anti-inflammatory cytokines and other mediators, mainly from skeletal muscle cells known as myokines (Leal et al., 2018), including IL-10 and IL-1 receptor antagonist (IL-1ra) (Pedersen and Toft, 2000). These myokines interact with a variety of immune-metabolic mediators predominantly secreted by the adipose tissue (Leal et al., 2018). Studies have shown that physical activity stimulates anti-inflammatory response (Pedersen and Toft, 2000), increases insulin sensitivity, and glycogen synthesis in skeletal muscle cells (Tomas et al., 19852002). In addition, recent studies from our group have shown that sera from elite athletes differ from people with regular activity in many factors, metabolic and inflammatory, depending on the type and level of exercise, which has a direct effect on insulin signaling (Al-Khelaifi et al., 2018a; Al-Khelaifi et al., 2018b; Al-Khelaifi et al., 2019; Sellami et al., 2021a).

Given the well-established impact of exercise on reducing IR, and provided that metabolomic profiles in the sera of elite athletes (secretome) vary among different sports, the effect of sera from elite athletes with different endurance levels and power classes on cellular insulin signaling is expected but not yet fully investigated. This study aims to explore the impact of sera of elite athletes from different groups of sports on preadipocytes (represented by 3T3-L1 cells) and skeletal muscle cells (represented by C2C12 cells), focusing on secreted inflammatory cytokines and activation of insulin signaling molecules.

## Methods

### Study design

Participants of this experimental study include nine consenting elite male athletes from different sport disciplines (fencers, *n* = 3; road cyclists, *n* = 3; bodybuilders *n* = 3) who participated in national or international sports events and tested negative for doping use at the anti-doping laboratory in Italy. In addition, three consenting healthy non-athlete participants from Al Wakra hospital (BMI, 26–27 kg/m^2^) were included as controls. The age of all participants including the controls was matched between 21 and 31 years old. In brief, athletes’ blood samples were collected when they were out of competitions, and these were delivered on ice to the anti-doping labs within 36 h. Once received, samples were immediately centrifuged to separate the serum and then stored at −20°C until further analysis. Due to the strict anonymization process adopted at anti-doping laboratories and those dictated by the study’s ethics, the only information available to the researchers was the type of sport, participants’ age, and gender. Serum samples were transported on dry ice to Qatar University and stored at −80°C degrees until further analysis. According to previously published criteria (Mitchell et al., 2005; Baman et al., 2010; Al-Khelaifi et al., 2018a), athlete participants were divided into three groups based on the maximum oxygen uptake (VO_2_max) and maximal voluntary contraction (MVC) of their sports. The high-endurance (HE) athlete group involved road cyclists who have high V $O_{2}$ max, and the high-power (HP) group included bodybuilders who have high MVC, whereas the fencers’ sera were allocated to the low-endurance/low-power (LE/LP) group since fencing involves low MVC and V $O_{2}$ max (Mitchell et al., 2005). The control serum samples were obtained from Al Wakra hospital under the same transporting conditions. The study was conducted under the guidelines of the World Medical Association Declaration of Helsinki. All protocols were approved by the Institutional Research Board of Qatar University (QU-IRB 1277-E/20 and QU-IRB 1548-EA/21).

### Cell culture

Mouse 3T3-L1 preadipocytes (CL-173, ATCC, Manassas, United States) and C2C12 myoblast cell line (CRL-1772, ATCC, Manassas, United States) were cultured in DMEM growth media (Gibco, Thermo Fisher Scientific, Waltham, United States) supplemented with 10% heat-inactivated bovine calf serum (Sigma-Aldrich, Darmstadt, Germany), 1% antibiotics (Gibco), and 1% L-glutamine (Gibco). After reaching confluency, cells were seeded in a 24-well plate at a seeding density of 42, 0000/well. After 24 h, the medium was changed to DMEM growth media without serum for 24 h. Following cell’s starvation, cells were washed once with phosphate buffered saline (PBS) to remove any remnants of FCS and then treated with a conditioned media containing 10% human serum of one of the different athlete serum groups (LE/LP, HE, and HP) or the non-athlete human serum (*n* = 3 per group) as a negative control. Conventional fetal bovine serum (FBS)-containing media was also used as a control for cell culture. After 72 h incubation, media supernatants were collected from all wells for measurement of cytokines, and the cells were lysed using RIPA buffer (Thermo Fisher Scientific) to measure insulin signaling proteins’ phosphorylation.

### Cytokine profiling

After growing 3T3-L1 and C2C12 cells for 72 h under the four culture conditions (supplementation with 10% serum from HP, HE, and LP/LE athletes or non-athlete control), media were collected to measure the concentration of secreted (mouse-specific) cytokines from the treated cells in order to evaluate the inflammatory status triggered by these interventions. Accumulated levels of six secreted mice cytokines (IFN gamma, IL-1 beta, IL-6, IL-10, IL-17A (CTLA-8), and TNF-alpha) in the media supernatants were measured using the inflammatory cytokine ProcartaPlex™ mouse and rat mix and match 6-plex panels (PPX-06-MXXGTDN, Thermo Fisher Scientific) using the Luminex™ 200 analyzer according to the manufacturer’s instructions (Luminex, Madison, WI, United States). Data were analyzed using xPONENT 4.2 software (Luminex™ 200 analyzer, Luminex, Madison, WI, United States).

### Insulin signaling

The change in phosphorylation of insulin signaling biomarkers [p70S6K (Thr412), IRS1 (Ser636), GSK3-α (Ser21), GSK3-β (Ser9), Akt (Ser473), PTEN (Ser380), IR (Tyr1162/Tyr1163), IGF1R (Tyr1135/Tyr1136), RPS6 (Ser235/Ser236), TSC2 (Ser939), and mTOR (Ser2448)] was quantified in lysates containing equal concentration of proteins (25 μg) prepared from 3T3-L1 and C2C12 cells using an Akt/mTOR phosphoprotein 11-plex magnetic bead kit 96-well plate (48-611MAG, Millipore MILLIPLEX, United States), following the manufacturer’s instructions. Mean fluorescent intensity (MFI) was assessed using Luminex 200 using xPONENT 4.2 software (Luminex™ 200 analyzer, Luminex, Madison, WI, United States). Table 1 lists these proteins and summarizes their roles in insulin signaling (Gual et al., 2005; Jolivalt et al., 2008; Frosig and Richter, 2009; Zhang et al., 2009).

**TABLE 1:** Insulin signaling proteins and the role of their activation (phosphorylation) in insulin signaling cascade, arranged according to the physiological order of signal transduction from outside to inside the cell.

Signaling molecule	Role	Phosphorylation	Protein activity	Insulin signaling
IR	Insulin resistance	**⇑**	**⇑**	**⇑**
IGF1R	Receptor of IGF1 and insulin (with lower affinity)	**⇑**	**⇑**	**⇑**
IRS-1	The first insulin signal transductor	**⇑**	**⇑**	**⇑**
PTEN	Negative regulator of IRS1/2 and PIP3	**⇑**	**⇑**	**⇓**
GSK3-β	Negative regulator (inhabits) of glycogen synthesis	**⇑**	**⇓**	**⇑**
GSK3-α	Negative regulator (inhabits) of glycogen synthesis	**⇑**	**⇓**	**⇑**
Akt	Inhibitor of AS160, TSC2, and GSK3 (all are inhibitors of downstream activities of insulin)	**⇑**	**⇑**	**⇑**
TSC2	Negative regulator (inhibitor) of mTOR	**⇑**	**⇓**	**⇑**
mTOR	Adipocyte differentiation and activation of p70s6k	**⇑**	**⇑**	**⇑**
p70s6k	Negative regulator of IRS1/2	**⇑**	**⇑**	**⇓**
RPS6	Facilitating mRNA translation and protein synthesis	**⇑**	**⇑**	**⇑**

The downward arrows indicate the decrease in protein activity or insulin sensitivity (negative regulation of insulin signaling), whereas the upward arrows represent the increase in protein activity or insulin sensitivity (positive regulation of insulin signaling).

### Data analysis

The results were statically analyzed using one-way ANOVA followed by independent sample *t*-test with (SPSS, IBM, and USA) software to examine the significant difference between the sample groups mean and the serum control mean. Data were presented as mean ± standard deviation (SD) in bar graphs and heatmap and mean ± SEM in figures.

## Results

### Effect of athletes’ sera on induction of pro-inflammatory cytokines

**FIGURE 1:**
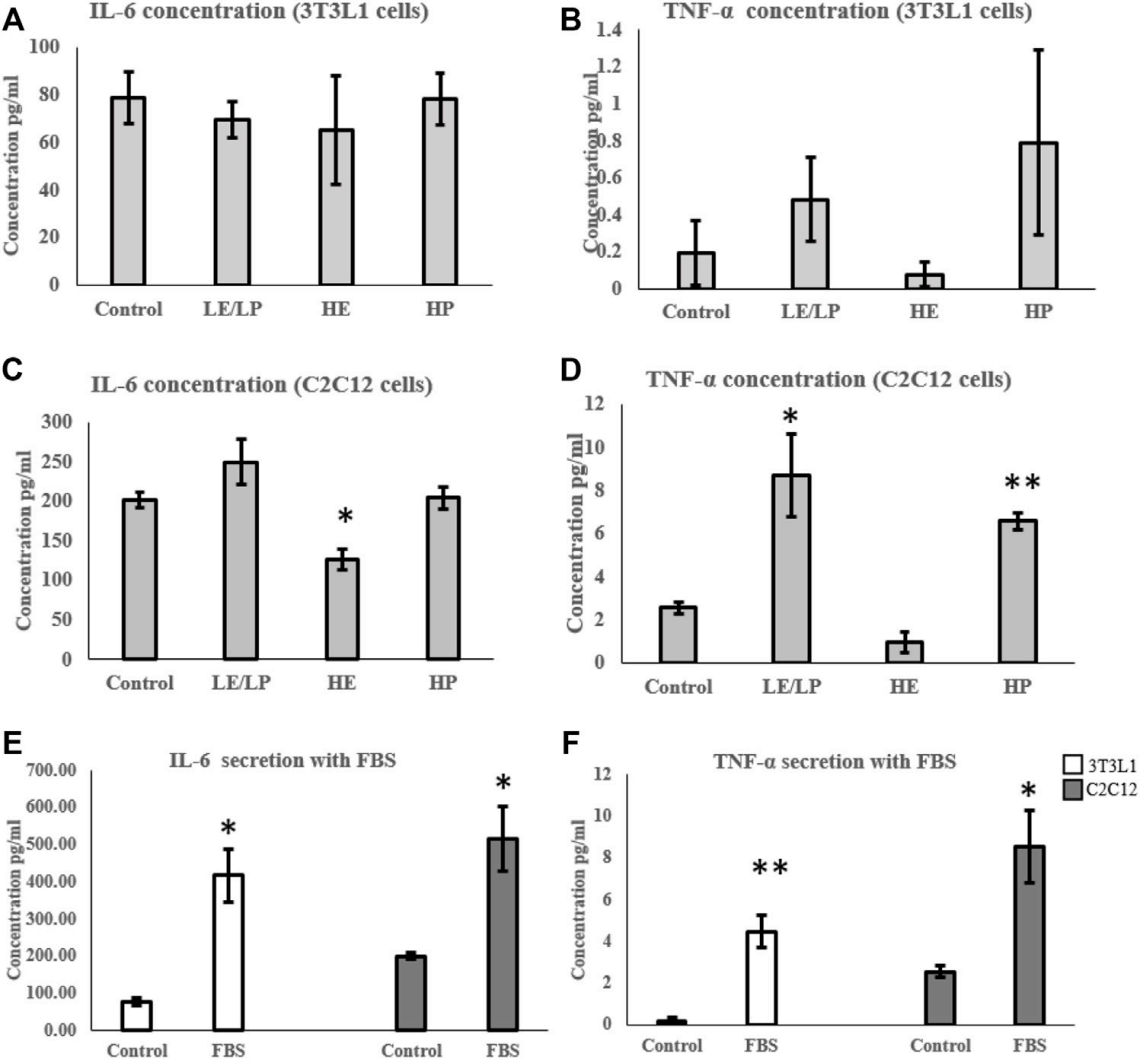
Effect of incubating 3T3-L1 **(A,B)** and C2C12 **(C,D)** cells with 10% of sera from different athlete groups (LE/LP: low endurance/low power, HE: high endurance, and HP: high power) on IL-6 and TNF-α secretion. The effect of incubating 3T3-L1 and C2C12 cells with FBS on the secretion of IL-6 **(E)** and TNF*-α* **(F)**. Data are presented as mean ± SEM (*p ≤ 0.05, **p ≤ 0.01).

Cytokine profiling of conditioned media of cells incubated with 10% human serum from one of the four study groups (LE/LP, HP, and HE, or non-athlete control) showed significant changes in levels of secreted IL-6 and TNF-α. Other cytokines were either absent or below the level of detection. 3T3-L1 cells incubated with the three athlete sera groups showed no significant variation in the secreted IL-6 (Figure 1A) or TNF-α (Figure 1B) compared with the non-athlete control serum. Conversely, C2C12 cells incubated with the three athlete sera groups showed significant differences in the levels of IL-6 and TNF-α depending on the type of sport. Whereas HE athlete serum exhibited an anti-inflammatory effect in the C2C12 cells represented by the significant decrease in IL-6 (0.4-fold decrease, *p* = 0.015) (Figure 1C), and there was a trend of decrease in TNF-α concentration (*p* = 0.06) (Figure 1D) compared to the negative control; both LE/LP and HP athlete sera groups exhibited a pro-inflammatory effect manifested by the significant increase in secreted TNF-α concentration (2-folds, *p* = 0.04) with serum from the LE/LP group, and 1.5-fold increase (*p* = 0.001) with serum from HP group (Figure 1D) compared to the negative control. Surprisingly, when cells were incubated with conventional FBS, both secreted IL-6 and TNF-α increased significantly in both cell lines. Accordingly, IL-6 secretion increased 5-fold in 3T3-L1 (*p* ≤ 0.05) and 2.6-fold in C2C12 (*p* ≤ 0.05) (Figure 1E), whereas TNF-α increased 22.5-fold in 3T3-L1 (*p* ≤ 0.01) and 3.3-fold in C2C12 (*p* ≤ 0.05) cells (Figure 1F) compared to human non-athlete control, suggesting a pro-inflammatory effect of FBS in both cell lines.

### The effect of athletes’ sera on insulin signaling in 3T3-L1 cells

**FIGURE 2:**
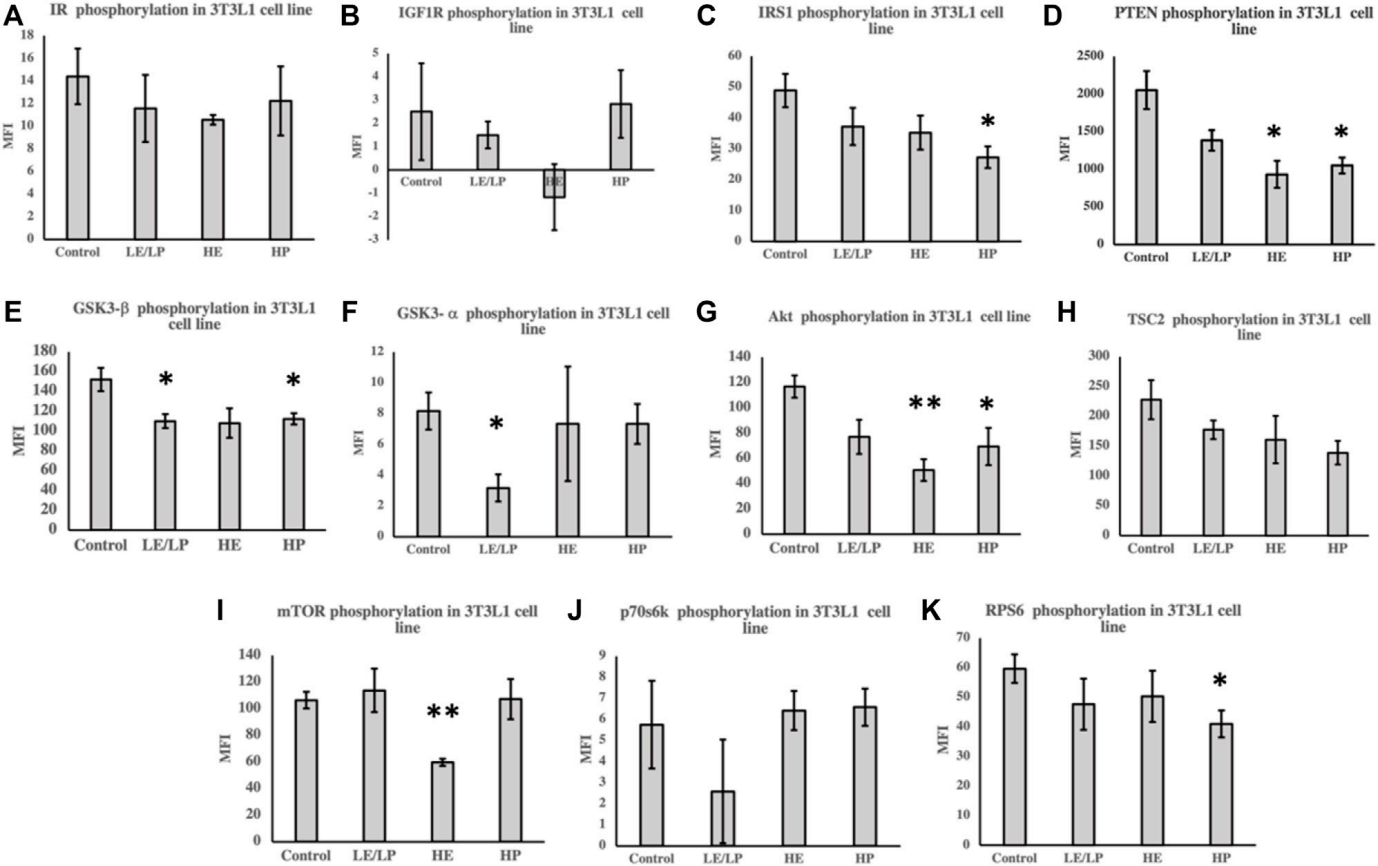
Effect of incubating 3T3-L1 cells with 10% of sera from different athlete groups (LE/LP: low endurance/low power, HE: high endurance, and HP: high-power and non-athlete control) on the activation of insulin signaling phosphoproteins, including insulin resistance (IR) **(A)**, insulin-like growth factor 1 receptor (IGF-1R) **(B)**, insulin receptor substrate 1 (IRS-1) **(C)**, phosphatase and tensin homolog deleted on chromosome 10 (PTEN) **(D)**, glycogen synthase kinase 3 beta (GSK3-β) **(E)**, glycogen synthase kinase 3 alpha (GSK3-α) **(F)**, protein kinase B (Akt) **(G)**, tuberous sclerosis complex 2 (TSC2) **(H)**, mammalian target of rapamycin (mTOR) **(I)**, ribosomal protein S6 kinase beta-1 (p70S6K) **(J)**, and ribosomal protein S6 (RPS6) **(K)**. Data are presented as mean ± SEM (*p ≤ 0.05, **p ≤ 0.01).

In order to assess the effect of different athlete sera on insulin signaling in preadipocytes and myoblasts, the activity of different components of insulin signaling was measured in the lysates prepared from 3T3-L1 cells supplemented with 10% serum from the four studied groups. Incubation of 3T3-L1 cells with sera from LE/LP, HE, and HP athlete groups decreased the phosphorylation of several insulin signaling proteins (Figures 2A–K). Cells incubated with serum from HP group showed decreased phosphorylation of IRS-1 by 40% (*p* = 0.02) (Figure 2C), PTEN by 40% (*p* = 0.02) (Figure 2D), GSK3-β by 30% (*p* = 0.03) (Figure 2E), Akt by 60% (*p* = 0.04) (Figure 2G), and RPS6 by 30% (*p* = 0.04) (Figure 2K) compared to non-athlete serum control. Similarly, there was a significant decreased phosphorylation of both GSK3-β (30%, *p* = 0.03) (Figure 2E) and GSK3-α (60%, *p* = 0.02) (Figure 2F) in preadipocytes incubated with the LE/LP serum group compared to non-athlete serum control. Furthermore, incubation with HE serum caused a significant decrease in the phosphorylation of PTEN by 50% (*p* = 0.02) (Figure 2D), Akt by 50% (*p* = 0.01) (Figure 2G), and mTOR by 40% (*p* = 0.002) (Figure 2I) compared to the negative control. Moreover, a trend of non-significant decrease in the phosphorylation of IR (*p* = 0.1) (Figure 2A), IRS-1 (*p* = 0.1) (Figure 2C), and GSK3-β (*p* = 0.08) (Figure 2E) was observed compared to the non-athlete control.

### The effect of athletes’ sera on insulin signaling in C2C12 cells

**FIGURE 3:**
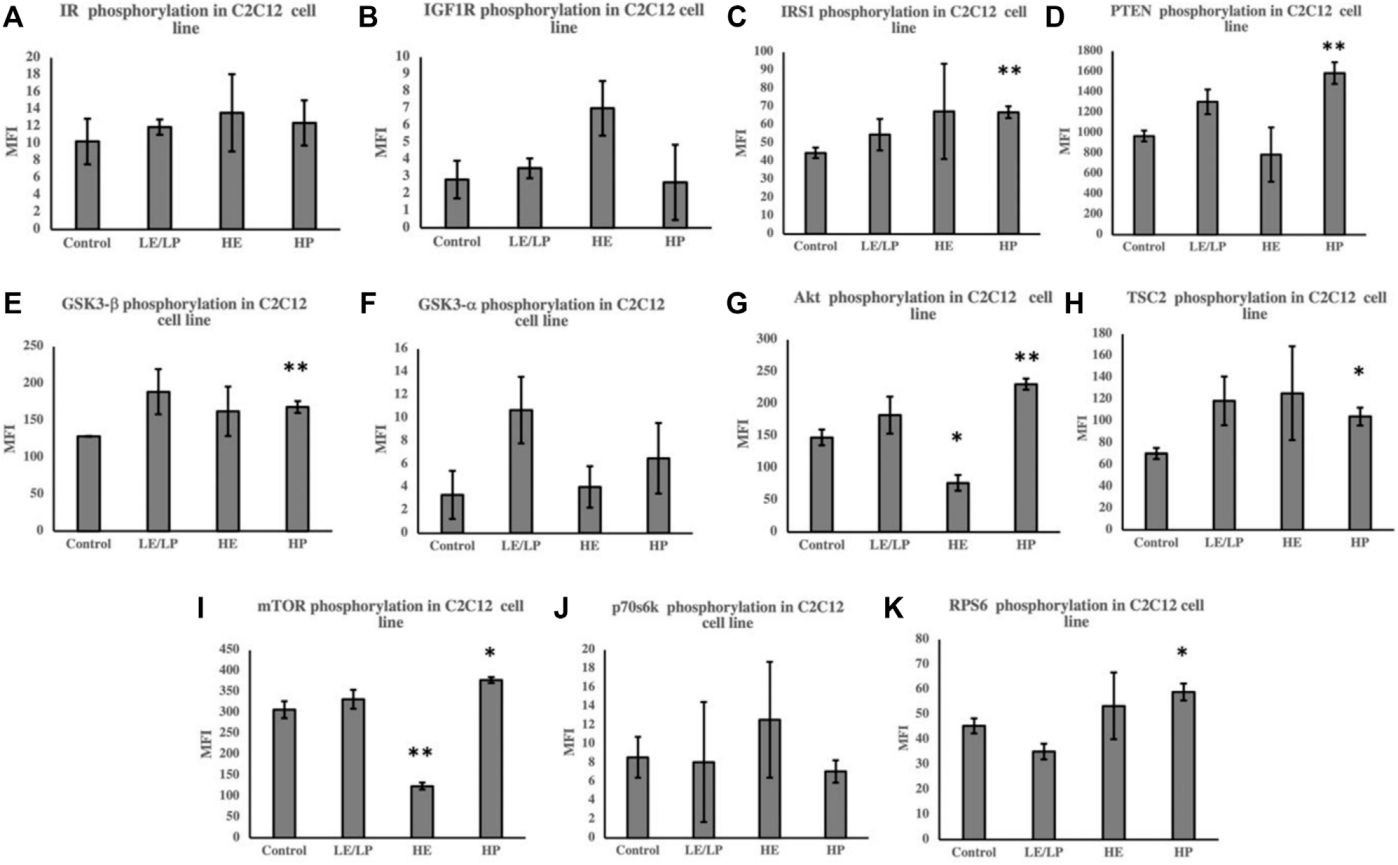
Effect of incubating C2C12 cells with 10% of sera from different athlete groups (LE/LP: low endurance/low power, HE: high endurance, and HP: high power) on the activation of insulin signaling phosphoproteins, including insulin resistance (IR) **(A)**, insulin-like growth factor 1 receptor (IGF-1R) **(B)**, insulin receptor substrate 1 (IRS-1) **(C)**, phosphatase and tensin homolog deleted on chromosome 10 (PTEN) **(D)**, glycogen synthase kinase 3 beta (GSK3-β) **(E)**, glycogen synthase kinase 3 alpha (GSK3-α) **(F)**, protein kinase B (AKT) **(G)**, tuberous sclerosis complex 2 (TSC2) **(H)**, mammalian target of rapamycin (mTOR) **(I)**, ribosomal protein S6 kinase beta-1 (p70S6K) **(J)**, and ribosomal protein S6 (RPS6) **(K)**. Data are presented as mean ± SEM (*p ≤ 0.05, **p ≤ 0.01).

Unlike 3T3-L1 cells, incubation of C2C12 cells with serum from HP athletes caused an increase in the phosphorylation of several insulin signaling molecules (Figures 3A–K), including IRS1 (50%, *p* = 0.006) (Figure 3C), PTEN (60%, *p* = 0.006) (Figure 3D), GSK3beta (30%, *p* = 0.007) (Figure 3E), Akt (60%, *p* = 0.005) (Figure 3G), TSC2 (50%, *p* = 0.02) (Figure 3H), mTOR (20%, *p* = 0.03) (Figure 3I), and RPS6 (30%, *p* = 0.04) (Figure 3K). HE serum, on the other hand, caused decreased phosphorylation of Akt (Figure 3G) and mTOR (Figure 3I) by 50% (*p* = 0.01 and *p* = 0.001, respectively).

**FIGURE 4:**
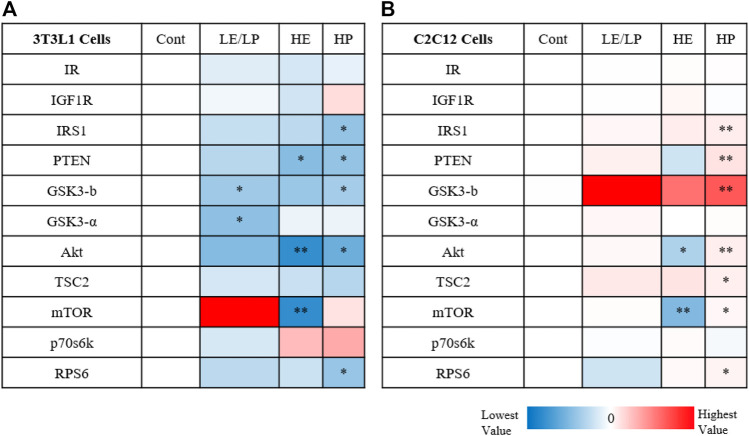
Heatmap illustrating the activity of insulin signaling phosphoproteins in response to 10% of sera from different athlete groups (LE/LP: low endurance/low power, HE: high endurance, and HP: high power) in 3T3-L1 cells **(A)** and C2C12 **(B)**. The dark red color marks the highest increase in phosphoprotein activity between the treatment (serum group) and the control, while the deep blue color represents the lowest decrease in phosphoprotein activity between the treatment (serum group) and control. Statistical significance was determined by one-way ANOVA followed by independent sample *t*-test (*p ≤ 0.05, **p ≤ 0.01).

Comparing the activity of insulin signaling-related phosphoproteins in response to sera from different athlete groups between 3T3-L1 and C2C12 suggests profound differences in their mode of action, especially with serum from the HP group (Figure 4). While serum from HP causes inhibition of insulin signaling in 3T3-L1 (Figure 4A), it also induces insulin signaling in C2C12 (Figure 4B).

## Discussion

Previous studies have shown that acute short-term exercise can trigger a pro-inflammatory effect presented by elevated secretion of IL-6 and CRP, and to a lesser extent, TNF-α levels, in addition to triggering leukocytosis and oxidative stress (Pedersen and Toft, 2000; Khazaei, 2012; Peake et al., 2015). Conversely, moderate regular exercise exhibits the opposite effect by inducing a marked anti-inflammatory status where the initial period of post-exercise elevation of pro-inflammatory cytokines (IL-6, TNF-α, and IL-1β) is followed by increased regulatory or anti-inflammatory myokines and cytokines, including IL-1ra, IL-4, IL-13, and IL-10 (Pedersen and Toft, 2000; Cerqueira et al., 2019). Excessive exercise in elite athletes from different classes of sports could modulate immune-inflammatory response differently (Sohail et al., 2020). Since inflammation is one of the underlying mediators of IR, exercise-associated immune adaptation might induce or reduce IR (Pedersen and Toft, 2000; Zierath, 2002; Wilund, 2007; Dimitrov et al., 2017). Moreover, the secretome of elite athletes, including the circulating cytokines, is found to differ among athletes with different endurance levels and power sports (Sohail et al., 2020). However, the impact of this variability on cellular insulin signaling and the secreted myokines and adipokines is less described. Thus, the current study examined and compared the secreted cytokines and activation of insulin signaling in preadipocytes and myoblasts in response to three athlete sera groups compared with the non-athlete human serum control. Our data reveal that the level of pro-inflammatory cytokines secreted from preadipocytes and myoblasts, and the modification of insulin signaling in these cells, varied when these cells are incubated with sera from different athlete groups. The variable response could reflect the impact of circulating cytokines and other factors in athletes who play sports that differ in their duration, intensity, and degree of associated muscle injury.

In this study, sera from all groups did not have a significant effect on cytokine secretion in 3T3-L1 preadipocytes compared to non-athlete serum. However, skeletal muscle cells treated with sera from LE/LP and HP athletes exhibited higher TNF-α, suggesting induction of a pro-inflammatory phenotype, whereas serum from HE athletes showed lower IL-6 secretion, suggesting a reduced pro-inflammatory phenotype. Although IL-6 is considered anti-inflammatory in the context of acute phase myokines secreted from skeletal muscle, reduced secretion from C2C12 should be considered pro-inflammatory; however since it was triggered by serum from HE athletes under chronic inflammatory status associated with chronic muscle contraction (Sellami et al., 2021b), its reduction was considered anti-inflammatory in this case. HP elite athlete training involved acute muscular damage, which induced rapid inflammatory cytokines secretion, namely, IL-6 and TNF-α by skeletal muscles. Previous findings by Sohail et al. (2020) indicated that HP sports were associated with a remarkable oxidative stress and pro-inflammatory profile, potentially explaining the present findings on skeletal muscle cells. The higher secretion of TNF-α in LE/PE could also reflect the acute muscle damage associated with fencing sports (Chen et al., 2017), although this requires further investigation. In contrast, serum from HE athletes was found to promote an anti-inflammatory response by skeletal muscle cells as demonstrated by the reduction of both pro-inflammatory cytokine’s secretion, compared to the non-athlete group. This is consistent with previous studies that showed that endurance training reduced IL-6 and TNF-α when compared to non-athletes, in addition to its effect on reducing the production of IL-6 mRNA in skeletal muscles (Petersen and Pedersen, 19852005; Fischer et al., 2004; Gokhale et al., 2007). Endurance exercise in elite endurance athletes might account for this finding as the body adapts to exercise-induced inflammation with regular exposure resulting in the anti-inflammatory status (Gokhale et al., 2007). Unexpectedly, both cell lines (3T3-L1 and C2C12) supplemented with conventional FBS-containing media showed a very significant inflammatory response. This finding suggested a pro-inflammatory effect of FBS, which sheds doubt on experiments run using FBS that might be stressing the cells and causing a pro-inflammatory background.

Given the strong association between inflammation of adipose tissue and skeletal muscles and development of IR, activity of insulin signaling molecules (Table 1) was assessed in 3T3-L1 and C2C12 cells supplemented with sera from different athlete groups. A general decrease in the phosphorylation of insulin signaling molecules was observed in 3T3-L1 cells incubated with different athlete serum treatments, suggesting reduced insulin sensitivity. Conversely, examination of insulin signaling in C2C12 cells revealed an overall increase in phosphorylation of insulin signaling molecules in comparison with non-athletes serum. LE/LP athlete serum seemed to reduce insulin sensitivity in preadipocytes (3T3-L1) compared with the non-athlete control, as manifested by the significant decrease in phosphorylation of GSK3-β and GSK3-α, while in skeletal muscle cells, despite the lack of statistical significance, it seemed to have a trend of increased insulin signaling since it increased the phosphorylation of almost all insulin signaling pathway related proteins. The HP athlete serum was found to manifest the same effect, though with higher significance, where the decrease of GSK3-β, IRS1, Akt, and RPS6 phosphorylation in preadipocytes was correlated with the reduction of insulin signaling in these cells compared to the non-athlete control. Like the LE/LP athlete serum, HP athlete serum appeared to increase insulin sensitivity in skeletal muscle cells presented by the significant increase in GSK3-β, IRS1, Akt, mTOR, and TSC2 phosphorylation. The effect of LE/LP and HP athlete serum on insulin signaling of preadipocytes and skeletal muscles were in agreement with the former investigations of exercise-associated insulin signaling (Kwak, 2013; Richter et al., 2021). During exercise, skeletal muscles are the main consumers of energy, while the adipocytes (major sites of fat storage) work to hydrolyze triglycerides to produce energy (Kwak, 2013). Since the primary sources of energy during exercise are carbohydrates and fat, insulin is needed to facilitate carbohydrates entry into the working skeletal muscles to be utilized. Therefore, insulin sensitivity tends to increase in skeletal muscles and decrease in adipocytes to suppress insulin-induced energy storage. In terms of intensity, during low-intensity exercise, the extracellular domains from blood are the main supplier for muscles energy metabolism, whereas the internal muscular energy stores plays a major role in high-intensity exercises. During the first hour of exercise, the plasma FFA and blood glucose are the second sources of energy, and as the duration increases the internal stores decline and the external sources become predominant (van Loon et al., 2001). In correlation with our data, compared to sedentary serum, insulin signaling in preadipocytes was reduced with all athlete sera as expected. The LE/LP athlete training is usually less prolonged with low intensity (i.e., their muscles utilize blood glucose to a lesser amount); thus, their serum slightly induced insulin signaling in skeletal muscle cells compared to non-athlete serum. While in HP sports, athletic training such as bodybuilding tends to involve a large muscular force for more than 1 h, resulting in an increased demand of energy consumption by the muscles from internal and external sources, and this was reflected by having the most significant increase of insulin signaling in skeletal muscles treated with their serum, and the most significant decrease in preadipocytes.

Unexpectedly, we found that HE athlete serum seems to decrease the insulin sensitivity in both preadipocytes and myoblasts, represented by the significant decrease in Akt and mTOR phosphorylation in 3T3-L1 and C2C12 cells in comparison to the control. The decrease of insulin signaling in preadipocytes was in alignment with the previously explained role of these cells during physical activity as an energy provider, yet the decrease in skeletal muscle cells was not theoretically anticipated. Nevertheless, Frosig et al. (2007) concluded that the improvement of insulin-stimulated glucose uptake after endurance training was not related to improvement in insulin signaling, but rather from hemodynamic adaptation of the body. Furthermore, elite cyclists’ endurance training programs might place them in the overtraining status that could lead to impairment of insulin signaling in skeletal muscles as stated by Pereira et al. (2016).

Taken together, inflammation induced by pro-inflammatory cytokines such as IL-6 and TNF-α is inversely correlated with insulin signaling where it might lead to insulin resistance (Table 2). This inhibitory effect of IL-6 and TNF-α on insulin signaling is mainly accomplished by inhibiting insulin resistance and IRS 1,2 phosphorylation, and GLUT-4 translocation (Papaetis et al., 2015). However, there are molecules other than cytokines in serum that can reduce insulin signaling including FFAs, lipotoxicity, hyperglycemia, and hyperinsulinemia (Khalid et al., 2021). In our study, it appears that inflammation contributed to reduced insulin signaling in preadipocytes treated with LP/LE and HP athlete sera as manifested by the increased inflammation and decreased insulin signaling with reduction of insulin resistance and IRS-1 phosphorylation post-treatment. In contrast, the same athlete serum groups (LE/LP and HP) also increased the inflammation in skeletal muscle cells but insulin signaling was rather increased, suggesting the interference of other serum factors that prevent the effect of IL-6 and TNF-α on insulin sensitivity. HE serum, on the other hand, showed a discrepant result between inflammation and insulin signaling, where they were both increased in the two cell types (preadipocytes and skeletal muscle cells). One possible cause of this discrepancy in skeletal muscles, where high endurance exercise is expected to enhance insulin signaling, is that this improvement declines after a few days of exercise cessation, and since our samples were obtained from athletes out of competition with no information about the duration between sample collection and last session of exercise, it could be an interfering factor (Keshel and Coker, 2015).

**TABLE 2:** Summary of changes in secreted cytokines and insulin activation in preadipocytes (3T3-L1) and progenitor skeletal muscle cells (C2C12) treated with 10% of sera from different athlete groups (LE/LP: low endurance/low power, HE: high endurance, and HP: high power).

Cell line	Athlete serum	Cytokines profiling	Insulin signaling
3T3-L1	LE/LP	⇑	⇓
(preadipocytes)	HE	⇓	⇓
	HP	⇑	⇓
C2C12	LE/LP	⇑	⇑
(myoblasts)	HE	⇓	⇓
	HP	⇑	⇑

One of the major limitations of our study is the low number of samples per group, despite the initial number of participants, matching the athlete’s gender, age, and their corresponding sports to avoid possible confounders has minimized the sample size; therefore, further studies are needed to confirm these findings. Another important limitation is the lack of assessment of initial cytokine concentrations in the serum samples before treatment, although our previous study has shown that the combined high-power sports showed greater oxidative stress and less anti-inflammatory profile than endurance sports (Sohail et al., 2020). Moreover, blood sample collection was not fully controlled; they were collected at different times, and the information about the period between sample collection and last training session was limited according to the strict regulations of anti-doping laboratories. However, according to anti-doping regulation, samples were acquired after 2 h of rest for biological passport to provide more deep data, yet a possible batch effect might have impeded the association between athlete serum classes and the study results of cytokine profile and insulin signaling. In addition, the relationship between physical activity and the immune response is complicated due to the interference of multiple individual variability parameters such as nutritional status, medications and supplements, individual athlete training frequency and intensity, and sleeping patterns, which is a known difficulty for studies in this field. Also, several serum components that affect insulin sensitivity might have confounded the interrelation between inflammation and impairment of insulin signaling, such as hyperinsulinemia, hyperglycemia, and the presence of red blood cells in some samples, where the insulin resistances on their surface could bind insulin instead of the studied cells (Zancan and Sola-Penna, 2005). Despite these limitations, this pilot study highlighted remarkable variations in insulin signaling and inflammatory response by preadipocytes and myoblasts among elite athletes’ serum treatment groups with sufficient power, yet larger studies would validate and confirm our findings.

In conclusion, this study has demonstrated that athlete serum from different sport disciplines exhibits different effects *in vitro* on the secretome and insulin signaling of preadipocytes and skeletal muscle cells and that this difference varies between the two cell lines. Compared to non-athlete human serum, LP/LE and HP athlete sera seem to exhibit an increased pro-inflammatory profile, and this immune response appears to affect insulin signaling in preadipocytes represented by decreased sensitivity, yet it was not linked with insulin sensitivity in skeletal muscle cells. In contrast, comparing the non-athlete serum with the HE athlete serum revealed the same effect in both cell lines having a decrease in the inflammatory biomarkers and insulin signaling simultaneously, suggesting the involvement of other IR contributors. The cellular source of changes in inflammatory cytokines is mainly attributed to acute muscular damage in athletes undergoing intense exercise regiments, which induces rapid inflammatory cytokines secretion by skeletal muscles. Changes in insulin sensitivity are probably secondary to inflammation although molecules other than cytokines in the serum can also reduce insulin signaling including FFAs, lipotoxicity, hyperglycemia, and hyperinsulinemia. These findings offer a better understanding of the complex relationship between different types of intense exercise in elite athletes and downstream inflammation and insulin resistance. Future studies will focus on translating these findings in athletes’ training programs to avoid excessive exercise-associated disorders and improve the use of physical activity as a potential intervention to modulate IR.

## Data Availability

The original contributions presented in the study are included in the article/Supplementary Material; further inquiries can be directed to the corresponding author.
